# Supplementary material for: Can changing neighborhoods influence mental health? An ecological analysis of gentrification and neighborhood-level serious psychological distress—New York City, 2002–2015
Source: PLoS One. 2023 Apr 5;18(4):e0283191. doi: 10.1371/journal.pone.0283191 (PMC10075454; doi:10.1371/journal.pone.0283191)
Supplement: S1 Table — (DOCX) [file pone.0283191.s001.docx]

**S1 Table. Neighborhood classification,** **New York City, 2000–2017**

| **Borough** | **Name** | **UHF #** | **Neighborhood median income in 2000^a^** | **Neighborhood average rent growth^b^** | **Neighborhood Type** |
| --- | --- | --- | --- | --- | --- |
| Bronx | Kingsbridge – Riverdale | 101 | Not Low income | ≤ median | Not Gentrifying |
| Bronx | Northeast Bronx | 102 | Not Low income | ≤ median | Not Gentrifying |
| Bronx | Fordham – Bronx Park | 103 | Low income | ≤ median | Not Gentrifying |
| Bronx | Pelham – Throgs Neck | 104 | Low income | > median but <100% | Gentrifying |
| Bronx | Crotona – Tremont | 105 | Low income | > median but <100% | Gentrifying |
| Bronx | High Bridge – Morrisania | 106 | Low income | > median but <100% | Gentrifying |
| Bronx | Hunts Point – Mott Haven | 107 | Low income | ≥100% | Hypergentrifying |
| Brooklyn | Greenpoint | 201 | Low income | ≥100% | Hypergentrifying |
| Brooklyn | Downtown – Heights – Slope | 202 | Not Low income | ≥100% | Not Gentrifying |
| Brooklyn | Bedford Stuyvesant – Crown Heights | 203 | Low income | ≥100% | Hypergentrifying |
| Brooklyn | East New York | 204 | Low income | > median but <100% | Gentrifying |
| Brooklyn | Sunset Park | 205 | Low income | > median but <100% | Gentrifying |
| Brooklyn | Borough Park | 206 | Low income | > median but <100% | Gentrifying |
| Brooklyn | East Flatbush – Flatbush | 207 | Low income | > median but <100% | Gentrifying |
| Brooklyn | Canarsie – Flatlands | 208 | Not Low income | ≤ median | Not Gentrifying |
| Brooklyn | Bensonhurst – Bay Ridge | 209 | Not Low income | > median but <100% | Not Gentrifying |
| Brooklyn | Coney Island – Sheepshead Bay | 210 | Low income | ≤ median | Not Gentrifying |
| Brooklyn | Williamsburg – Bushwick | 211 | Low income | ≥100% | Hypergentrifying |
| Manhattan | Washington Heights – Inwood | 301 | Low income | ≥100% | Hypergentrifying |
| Manhattan | Central Harlem – Morningside Heights | 302 | Low income | ≤ median | Not Gentrifying |
| Manhattan | East Harlem | 303 | Low income | ≥100% | Hypergentrifying |
| Manhattan | Upper West Side | 304 | Not Low income | ≤ median | Not Gentrifying |
| Manhattan | Upper East Side | 305 | Not Low income | ≤ median | Not Gentrifying |
| Manhattan | Chelsea – Clinton | 306 | Not Low income | ≤ median | Not Gentrifying |
| Manhattan | Gramercy Park – Murray Hill | 307 | Not Low income | ≤ median | Not Gentrifying |
| Manhattan | Greenwich Village – Soho | 308 | Not Low income | > median but <100% | Not Gentrifying |
| Manhattan | Union Square – Lower East Side | 309 | Not Low income | > median but <100% | Not Gentrifying |
| Manhattan | Lower Manhattan | 310 | Not Low income | ≤ median | Not Gentrifying |
| Queens | Long Island City – Astoria | 401 | Low income | ≥100% | Hypergentrifying |
| Queens | West Queens | 402 | Not Low income | ≤ median | Not Gentrifying |
| Queens | Flushing – Clearview | 403 | Not Low income | ≤ median | Not Gentrifying |
| Queens | Bayside – Little Neck | 404 | Not Low income | ≤ median | Not Gentrifying |
| Queens | Ridgewood – Forest Hills | 405 | Not Low income | > median but <100% | Not Gentrifying |
| Queens | Fresh Meadows | 406 | Not Low income | > median but <100% | Not Gentrifying |
| Queens | Southwest Queens | 407 | Not Low income | ≤ median | Not Gentrifying |
| Queens | Jamaica | 408 | Not Low income | ≤ median | Not Gentrifying |
| Queens | Southeast Queens | 409 | Not Low income | ≤ median | Not Gentrifying |
| Queens | Rockaway | 410 | Not Low income | > median but <100% | Not Gentrifying |
| Staten Island | Port Richmond | 501 | Not Low income | ≤ median | Not Gentrifying |
| Staten Island | Stapleton – St. George | 502 | Not Low income | ≤ median | Not Gentrifying |
| Staten Island | Willowbrook | 503 | Not Low income | ≤ median | Not Gentrifying |
| Staten Island | South Beach – Tottenville | 504 | Not Low income | ≤ median | Not Gentrifying |
| Data from 2000 Census and 2013–2017 American Community Survey (ACS) 5-year estimates  ^a^ Low income is <40th percentile for the city in the year 2000  ^b^ Neighborhood average rent growth was determined during 2000–2017 | | | | | |
