# Supplementary material for: Can changing neighborhoods influence mental health? An ecological analysis of gentrification and neighborhood-level serious psychological distress—New York City, 2002–2015
Source: PLoS One. 2023 Apr 5;18(4):e0283191. doi: 10.1371/journal.pone.0283191 (PMC10075454; doi:10.1371/journal.pone.0283191)
Supplement: Attachment — Submitted filename: PONE-D-21-39108_review.docx [file pone.0283191.s002.docx]

Title: Can changing neighborhoods influence mental health? An ecological analysis of gentrification and neighborhood-level serious psychological distress—New York City, 2002-2015

**Abstract:**

The abstract could better set up the argument for the paper by stating up front why there is an expected connection between gentrification and health equity.

The wording in the sentence starting “During 2000-2017” is a bit awkward. For instance, what is meant by “median and <=100% rent growth?

Is joinpoint intended to be capitalized?

**Introduction:**

The first two sentences seem a bit disjointed from the rest of the paper. It’s unclear how the neighborhoods experiencing poverty (does this mean neighborhoods with a relatively high percentage of residents with incomes under the poverty line, or neighborhoods where any residents are living in poverty?) is related to the next sentence about gentrification.

In the sentence starting “In many United States cities…”, it is odd to state that gentrification “builds upon” neighborhood disinvestment from redlining. Builds upon, has a positive connotation that I don’t think is deserved here.

The attribution of gentrification to historic redlining practices is and over simplification. Redlining was only one part of a long history of racist policies and practices that led to uneven urban development and segregation in cities. Therefore, I suggest changing the sentence starting with “due to redlining practices”.

The focus specifically on psychological distress seems sudden. Perhaps some of the studies reporting on mental health should be cited in the first paragraph rather than the study on self-rated general health.

What do you mean by “sporadic and small scale” in describing the first wave of gentrification?

Why do you choose the 1950’s as the date for the beginning of the first wave? This seems odd since the term gentrification wasn’t termed until the 1960’s.

I think more description of what was happening in the period from 2000-2017 in terms of trends in urban development and economic trends would be useful in making the case for studying this time period specifically.

When you say “finance-driven”, do you mean driven by investments from the financial industry, or something else?

It is not clear why the decision is made to focus specifically on hypergentrifying neighborhoods.

In the third paragraph, what is meant by increasing “physical infrastructure”? And what do you include as physical infrastructure?

It would be nice also to describe the hypothesized pathways that may exist for the relationship between gentrification and mental health distress. In addition to those cited, you might want to review these articles:

Andrew Binet, Gabriela Zayas del Rio, Mariana Arcaya, Gail Roderigues & Vedette Gavin (2021) ‘It feels like money’s just flying out the window’: financial security, stress and health in gentrifying neighborhoods, Cities & Health, DOI: [10.1080/23748834.2021.1885250](https://doi.org/10.1080/23748834.2021.1885250)

Anguelovski I, Cole HVS, O’Neill E, Baro F, Kotsila P, Sekulova F, Pérez del Pulgar C, Shokry G, Garcia Lamarca M, Arguelles Ramos L, Connolly JTT, Honey-Rosés J, Lopez Gay A, Fontán Vela M, Mathaney A, Oscilowicz E, Binet A,Triguero-Mas M. Gentrification pathways and their health impacts on historically marginalized residents in Europe and North America: Global qualitative evidence from 14 cities. *Health and Place*. 2021 Nov (Vol 72). <https://doi.org/10.1016/j.healthplace.2021.102698>.

**Methods:**

Should make clear why you decide to focus on hypergentrifying neighborhoods in the analysis (either here or in the introduction, or across both).

The dates included are confusing. It’s clear that the analysis couldn’t use the CHS data from 2017 since the mental health distress scale was not included, but it’s not clear why the census dates did not match the CHS data included (as in ending in 2015), especially since gentrification is the exposure of interest, and not the other way around. This is also confusing since the date 2015 is used in the title, but 2017 is used in other places.

Was the decision to use 13 as the cutoff for serious psychological distress based on some precedent or published source?

Could you describe a bit more what you mean in the sentence beginning “We analyzed 2015 data, …” What do you mean hear by contextualizing?

How was the census data aggregated into UHF areas?

As someone who does not know anything about joinpoint regression, I think it would be helpful if you could describe what joinpoint regression is and why you chose to use it?

**Results:**

In the section starting with “Demographics by level of gentrification”, and beyond, you refer to census data from 2000-2018 (instead of 2017 as stated earlier). Please clarify the years included.

In the “Demographics by level of gentrification” section, are the relative and absolute changes in demographics averaged over the years included, or do the represent change from 2000 to 2018, not considering the years in between?

**Discussion:**

The reasons why we might see the effects observed in hypergentrified neighborhoods but not gentrified ones should be better indicated and reasoned in the discussion.

In a few places, you use direct quotes from participants in other published studies. This seems odd to me.

In the paragraph starting “In our analyses…”, it might be useful to describe why you didn’t include length of residency as a criteria for being considered an “original resident”. In addition, you might state more clearly here why you use race/ethnicity as a proxy for being an original resident (which is implied, but could be more directly worded).

In the paragraph starting “health disparities are deeply entrenched in NYC…”, I find it confusing that you bounce between referencing studies from NYC and those from other places (like California). The purpose of the paragraph might be more clear if you refer only to NYC, and also state why NYC might be a unique case. There are other studies which are published about gentrification and health in NYC, for instance this one:

Cole HVS, Triguero-Mas M, Connolly J, Anguelovski I. Determining the Health Benefits of Green Space: Does gentrification matter? *Health and Place*. 2019; [57](https://www.sciencedirect.com/science/journal/13538292/57/supp/C), May 2019: 1-11. <https://doi.org/10.1016/j.healthplace.2019.02.001>

In the paragraph starting with “We observed different patterns of…”, it is a bit misleading to state that “… we have shown had a less extreme increase in rent growth…” since this is how you defined these neighborhoods and classified them in the first place, rather than a finding from the study.

In the paragraph starting with “An ecological study design inherently…”, you state that 2000 and 2018 are arbitrary dates. It might be more interesting then to also discuss and characterize what type of urban and economic development occurred during this time in NYC. Also, what effect do you think the 2008 recession had on your findings?

The focus on developing targeted interventions in the last paragraph is a bit misleading and I don’t think links well to the findings from the paper. The paper demonstrates the relationship between structural inequalities and mental health inequalities. But it’s unclear how and if the interventions planned by the health department could then develop interventions that address such structural inequalities, rather than to promote health interventions that merely target individuals or populations that are affected by such inequalities.

**Acknowledgements:**

Check the spelling of Kathleen Reilly’s name
