# Supplementary material for: Can changing neighborhoods influence mental health? An ecological analysis of gentrification and neighborhood-level serious psychological distress—New York City, 2002–2015
Source: PLoS One. 2023 Apr 5;18(4):e0283191. doi: 10.1371/journal.pone.0283191 (PMC10075454; doi:10.1371/journal.pone.0283191)
Supplement: Attachment — Submitted filename: Response to Reviewers.docx [file pone.0283191.s003.docx]

Dear Editor and Reviewer,

Thank you for the opportunity to revise and resubmit our manuscript titled “Can changing neighborhoods influence mental health? An ecological analysis of gentrification and neighborhood-level serious psychological distress—New York City, 2002-2015” and for the thoughtful feedback. We have included point-by-point response to all reviewer and editor comments below.

Sincerely,

Karen A. Alroy, DVM, MPH

NYC DOHMH Gotham Center

42-09 28th Street

Long Island City, NY 11101

Tel. 347.396.2917; Fax. 347.396.2753

Email: [kalroy@health.nyc.gov](mailto:kalroy@health.nyc.gov)

Haleigh Cavalier, MPH

NYC DOHMH Gotham Center

42-09 28th Street

Long Island City, NY 11101

Tel. 347.396.2917; Fax. 347.396.2753

Email: haleighcav23@gmail.com

**Reviewer #1**

**Abstract:**

The abstract could better set up the argument for the paper by stating up front why there is an expected connection between gentrification and health equity.

- Thank you for this point. We have modified the second and third sentences of the abstract to say “Gentrification is characterized by rapid development in historically disinvested neighborhoods. The burden of gentrification, including increased living expenses, and disrupted social networks, disproportionally impacts certain residents.”

The wording in the sentence starting “During 2000-2017” is a bit awkward. For instance, what is meant by “median and <=100% rent growth?

- Thank you for pointing this out. We have revised these sentences in the abstract to more clearly describe how neighborhoods were categorized. “We categorized New York City neighborhoods during 2000–2017 as hypergentrifying, gentrifying, and not-gentrifying using a modified New York University Furman Center index. Neighborhoods with >100% rent growth were hypergentrifying; neighborhoods with greater than median and ≤100% rent growth were gentrifying; and neighborhoods with less than median rent growth were not-gentrifying.”

Is joinpoint intended to be capitalized?

- Thank you for catching this. While the software name is Joinpoint (with a capital J), in the abstract here we are referring to Joinpoint regression (with a lowercase j).

**Introduction:**

The first two sentences seem a bit disjointed from the rest of the paper. It’s unclear how the neighborhoods experiencing poverty (does this mean neighborhoods with a relatively high percentage of residents with incomes under the poverty line, or neighborhoods where any residents are living in poverty?) is related to the next sentence about gentrification.

- Great point – the text has been updated to add clarity. It now reads: “This is particularly true regarding mental health, where social cohesion can confer strength, health, and resiliency. This social resiliency from neighborhood ties can benefit residents’ mental health, even among residents experiencing poverty or other hardship. However, social cohesion can be disrupted by gentrification (2, 3). Gentrification is the process of rapid neighborhood economic investment to repair and rebuild homes and businesses accompanied by an influx of middle-class or affluent people, who are often young and White (4).”

In the sentence starting “In many United States cities…”, it is odd to state that gentrification “builds upon” neighborhood disinvestment from redlining. Builds upon, has a positive connotation that I don’t think is deserved here.

- This is a great point. It has been changed from “builds upon” to “compounds”.

The attribution of gentrification to historic redlining practices is an over simplification. Redlining was only one part of a long history of racist policies and practices that led to uneven urban development and segregation in cities. Therefore, I suggest changing the sentence starting with “due to redlining practices”.

- Another great point. This has been changed and now reads: “In many United States cities, gentrification compounds decades of historic neighborhood disinvestment from longstanding racist policies and practices that have led to inequitable urban development. These include but are not limited to discriminatory homeownership practices like redlining, a financial lending practice that denied home ownership loans to low-income or minority populations (5), exclusionary zoning laws, and racial segregation of public housing (6). Thus, gentrification disproportionately affects racial minorities in highly segregated urban cities, exacerbating neighborhood inequality by race and class (7).”

The focus specifically on psychological distress seems sudden. Perhaps some of the studies reporting on mental health should be cited in the first paragraph rather than the study on self-rated general health.

- Thank you for this suggestion. We have included some description of existing literature regarding gentrification and mental health. We describe that “Gentrification has been identified as a perceived neighborhood stressor across diverse communities in NYC (9), and gentrification-related displacement has been associated with negative mental health outcomes (10).”

What do you mean by “sporadic and small scale” in describing the first wave of gentrification?

- We revised this by describing it as “highly-localized and small-scale”

Why do you choose the 1950’s as the date for the beginning of the first wave? This seems odd since the term gentrification wasn’t termed until the 1960’s.

- We revised this by describing the first wave of gentrification as “prior to the 1970s” as is consistent in the literature.

I think more description of what was happening in the period from 2000-2017 in terms of trends in urban development and economic trends would be useful in making the case for studying this time period specifically. When you say “finance-driven”, do you mean driven by investments from the financial industry, or something else?

- Thank you for these suggestions. We have added additional clarification about what is meant by finance-driven, and we also provided additional context as for why the later stage gentrification time period was the focus of this study. We wrote “Later-stage gentrification (the third through fifth waves) have been driven by money-making ventures and characterized by urban commodification (13). A recent analysis of NYC real estate indicated that in 2003 most building purchases were made by individuals, but by 2014 corporations represented the predominant property buyer in nearly all neighborhoods (14). Landlords that own multiple buildings, such as corporations, have a higher tenant eviction rate per unit compared with landlords who own few units (15).”

It is not clear why the decision is made to focus specifically on hypergentrifying neighborhoods.

In our methods section we describe that “to focus our analysis on the most pronounced manifestation of gentrification, we revised gentrifying neighborhoods into two more granular levels”

- We appreciate the point you’ve raised and added a paragraph in the discussion section, which further explores this categorization. We wrote “In our analysis we used three-tiers of neighborhood classification to examine gentrification. While both gentrifying and hypergentrifying neighborhoods had low median neighborhood income in the year 2000 and both neighborhood types experienced substantial rent increases, the demographic changes in gentrifying and hypergentrifying neighborhoods differed. With Census Bureau population estimate data from hypergentrifying neighborhoods, we found a decreased percentage of Black and Latino residents, and an increased percentage of people aged 25–44 years during 2000–2018. Similar demographic patterns were not observed in the neighborhoods classified as gentrifying.”

In the third paragraph, what is meant by increasing “physical infrastructure”? And what do you include as physical infrastructure?

- Thank you, this text has been clarified and now reads: “Gentrification can increase property value and lead to increases in and quality improvements of physical infrastructure such as grocery stores, green spaces, roads, restaurants and other service sector businesses.”

It would be nice also to describe the hypothesized pathways that may exist for the relationship between gentrification and mental health distress. In addition to those cited, you might want to review these articles:

Andrew Binet, Gabriela Zayas del Rio, Mariana Arcaya, Gail Roderigues & Vedette Gavin (2021) ‘It feels like money’s just flying out the window’: financial security, stress and health in gentrifying neighborhoods, Cities & Health, DOI: [10.1080/23748834.2021.1885250](https://doi.org/10.1080/23748834.2021.1885250)

Anguelovski I, Cole HVS, O’Neill E, Baro F, Kotsila P, Sekulova F, Pérez del Pulgar C, Shokry G, Garcia Lamarca M, Arguelles Ramos L, Connolly JTT, Honey-Rosés J, Lopez Gay A, Fontán Vela M, Mathaney A, Oscilowicz E, Binet A,Triguero-Mas M. Gentrification pathways and their health impacts on historically marginalized residents in Europe and North America: Global qualitative evidence from 14 cities. *Health and Place*. 2021 Nov (Vol 72). <https://doi.org/10.1016/j.healthplace.2021.102698>.

- Great suggestion. While we do not have the space for a full discussion of pathways, we have included the following sentence: “Proposed pathways through which gentrification could affect mental health include increased stress resulting from threats to housing and financial security, loss of institutional resources and services, and social as well as cultural displacement (19, 20).” And we have added the citations that you recommended – these are great papers!

**Methods:**

Should make clear why you decide to focus on hypergentrifying neighborhoods in the analysis (either here or in the introduction, or across both).

In the methods we added the clarification that “to focus our analysis on the most pronounced manifestation of gentrification, we revised gentrifying neighborhoods into two more granular levels”

- We have also added a paragraph in the discussion as mentioned above.

The dates included are confusing. It’s clear that the analysis couldn’t use the CHS data from 2017 since the mental health distress scale was not included, but it’s not clear why the census dates did not match the CHS data included (as in ending in 2015), especially since gentrification is the exposure of interest, and not the other way around. This is also confusing since the date 2015 is used in the title, but 2017 is used in other places.

- Thank you for raising this as a confusing point. We tried to provide additional clarification in the methods section. We wrote on page “Variables on serious psychological distress were not included in the 2017 Community Health Survey even though 2017 was the last year of ACS data that we used for neighborhood classification. To categorize neighborhood type we used small geographies, and consequently selected ACS 5-year data (2013-2017). The resulting two-year difference between ACS and Community Health Survey data sources, was considered acceptable since trend analysis and not annual data analysis was the focus of this work.”

Was the decision to use 13 as the cutoff for serious psychological distress based on some precedent or published source?

- Yes, the text has been changed to reflect this and two citations have been added that establish and use this cutoff.

Could you describe a bit more what you mean in the sentence beginning “We analyzed 2015 data, …” What do you mean hear by contextualizing?

- Yes, we have changed the wording for more clarity. The text now reads: “We further analyzed 2015 data, the most recent year this mental health indicator was measured, to understand differences in prevalence across different demographic groups.”

How was the census data aggregated into UHF areas?

- A sentence was added to address this at the end of the materials and methods section “classifying levels of gentrification.” It reads: “Data from the census are available at the Zip-Code Tabulation Area (ZCTA) geographic level and were aggregated to UHF by summing the values for each ZCTA within a given UHF.”

As someone who does not know anything about joinpoint regression, I think it would be helpful if you could describe what joinpoint regression is and why you chose to use it?

- Thank you for this suggestion. We have expanded on our description of joinpoint regression and Joinpoint software. For the analysis of trends over time we used a statistical software, known as Joinpoint (National Cancer Institute). The software uses joinpoint regression, also known as change point or segmented regression, to identify the presence or absence of segmented trends and inflection points, or joinpoints. Joinpoint software fits the simplest joinpoint model that the data allow. Although the software was developed for cancer trend analyses, Joinpoint has now been utilized for a diversity of analyses including studies on traffic regulations (28), and suicide rates (29).

**Results:**

In the section starting with “Demographics by level of gentrification”, and beyond, you refer to census data from 2000-2018 (instead of 2017 as stated earlier). Please clarify the years included.

- The 2017 5-year estimate ACS data were used to classify gentrification levels. The 5-year estimate data were recommended over 1-year or 3-year estimate data since the geography we used was smaller and the 5-year estimate data increase precision. At the time the paper was written, the 2017 data were the most recent of available data sets. The 2018 census population estimate data was used when looking at demographic variables and how they changed over time. We always used the most recent data that were available and although both data sets came from the Census Bureau, they are released at different times. Since the population estimates are released annually, these 2018 data were available at the time of writing. The language in the paper has been changed to reflect more clearly that the two data sets for these analyses are distinct. Data used in the demographics analysis are now always referred to as Bureau population estimates and data used in the gentrification analysis are referred to as 2000 Census/2017 ACS data. We decided to use 2018 data for the demographic analysis as the goal of this ancillary analysis was capture a demographic snapshot at the start and end of the period of gentrification we are focused on in the main analysis and compare the two.

In the “Demographics by level of gentrification” section, are the relative and absolute changes in demographics averaged over the years included, or do the represent change from 2000 to 2018, not considering the years in between?

- Thank you, upon re-reading this text, we realize “percent change” is confusing and not fully accurate. We used 2000 and 2018 data to look at absolute changes in the percent composition of various age and racial/ethnic groups. For example, if 30% of the population was Black in 2000 and 35% of the population was Black in 2018, the absolute change was +5%. The text in has been updated for clarity and now reads in the Methods Section: “In order to characterize the differences in hypergentrifying and gentrifying neighborhoods and to assess the changing demographics in each level of gentrification, we examined the absolute change in percent composition of age and racial/ethnic groups using Census Bureau annual population estimates from 2000 and 2018 (34).” And in the Results section: “According to the U.S Census Bureau population estimates from 2000 and 2018 in hypergentrifying neighborhoods, there was an increase in the percentage and total number of people who identified as White…”

**Discussion:**

The reasons why we might see the effects observed in hypergentrified neighborhoods but not gentrified ones should be better indicated and reasoned in the discussion.

- We have added to and emphasized the discussion of this topic in the paper. The text now reads: “What might be happening in the neighborhoods we classified as gentrifying but not hypergentrifying? Within UHF heterogeneity is one possible explanation. Displacement and gentrification maps created by the Urban Displacement Project suggest that the areas overlapping with the seven UHF neighborhoods we classified as gentrifying are comprised of a variety of different census tracts that are experiencing ongoing gentrification, are at risk of gentrification, are experiencing ongoing displacement of low-income households, and represent some tracts that are not losing low-income households (59). Also, gentrifying and hypergentifying categories may capture gentrification of a different magnitude or stage, which relate to the observed differences in health outcomes. This explanation is supported by the demographic differences observed in hypergentrifying and gentrifying neighborhoods. Community-based participatory research could offer additional qualitative data describing the experiences of residents in these neighborhoods and could help inform interpretation of the mental health outcomes we observed overall and by race/ethnicity in gentrifying neighborhoods.”

In a few places, you use direct quotes from participants in other published studies. This seems odd to me.

- This is understandable. We feel as though incorporating quotes from qualitative research studies in the discussion provides relevant context and highlights community opinions and voices on the topic, since we were not able to supplement this work with our own independent qualitative research.

In the paragraph starting “In our analyses…”, it might be useful to describe why you didn’t include length of residency as a criteria for being considered an “original resident”. In addition, you might state more clearly here why you use race/ethnicity as a proxy for being an original resident (which is implied, but could be more directly worded).

- Thank you for this feedback. We have acknowledged why it was difficult to assess who was an “original resident” using the Community Health Survey data available for this analysis. We wrote the following: “In our analyses, we hypothesized that most of the Black and Latino population in hypergentrifying neighborhoods were original residents and not new residents moving in during the gentrification process. Since Community Health Survey data are annual cross-sectional, and not longitudinal data, it was not possible to identify original residents. Using an approximation, we gained insight into the respondents’ residential mobility by examining how long people had lived in their home or apartment. We found that in 2017 in hypergentrifying neighborhoods, there were a higher proportion of Black and Latino residents who had lived in their homes for ≥5 years compared with White residents.”

In the paragraph starting “health disparities are deeply entrenched in NYC…”, I find it confusing that you bounce between referencing studies from NYC and those from other places (like California). The purpose of the paragraph might be more clear if you refer only to NYC, and also state why NYC might be a unique case. There are other studies which are published about gentrification and health in NYC, for instance this one:

Cole HVS, Triguero-Mas M, Connolly J, Anguelovski I. Determining the Health Benefits of Green Space: Does gentrification matter? *Health and Place*. 2019; [57](https://www.sciencedirect.com/science/journal/13538292/57/supp/C), May 2019: 1-11. <https://doi.org/10.1016/j.healthplace.2019.02.001>

- Thank you for this suggestion. We have refined the topic sentence to say “Health disparities are deeply entrenched in the United States (1, 47, 48), and this is not the first study to illustrate gentrification exacerbating health disparities.” In addition, we have clarified that even the benefits associated with green space in gentrifying neighborhoods is largely unequitable. “Even the benefits of active green space in gentrifying neighborhoods is not equitably shared among community members, but rather benefits are largely enjoyed by residents who are highly educated and have high incomes (51).”

In the paragraph starting with “We observed different patterns of…”, it is a bit misleading to state that “… we have shown had a less extreme increase in rent growth…” since this is how you defined these neighborhoods and classified them in the first place, rather than a finding from the study.

- Thank you. We have changed the wording. This sentence now reads “We observed different patterns of serious psychological distress in gentrifying neighborhoods, as compared with hypergentrifying neighborhoods, which experienced less extreme increase in rent growth and lacked corresponding demographic changes.”

In the paragraph starting with “An ecological study design inherently…”, you state that 2000 and 2018 are arbitrary dates. It might be more interesting then to also discuss and characterize what type of urban and economic development occurred during this time in NYC. Also, what effect do you think the 2008 recession had on your findings?

- In the introduction we discuss different types of urban and economic development and we describe the patterns of gentrification in NYC. We write “Scholars have described distinct waves of gentrification in NYC, and in other cities, that have occurred in the past century. The first wave, prior to the 1970s, was highly-localized and small-scale; the second wave, in the 1970s–1989, was linked with deindustrialization, a back-to-the-city movement, and was highly correlated with artist communities (11, 12). Later-stage gentrification (the third through fifth waves) have been driven by money-making ventures and characterized by urban commodification (13). A recent analysis of NYC real estate indicated that in 2003 most building purchases were made by individuals, but by 2014 corporations represented the predominant property buyer in nearly all neighborhoods (14). Landlords that own multiple buildings, such as corporations, have a higher tenant eviction rate per unit compared with landlords who own few units (15). This study focuses on neighborhoods that experienced later-stage gentrification occurring during 2000–2017.” Additionally, we did not observe any joinpoints associated with the 2008 recession indicating a change in the existing trend.

The focus on developing targeted interventions in the last paragraph is a bit misleading and I don’t think links well to the findings from the paper. The paper demonstrates the relationship between structural inequalities and mental health inequalities. But it’s unclear how and if the interventions planned by the health department could then develop interventions that address such structural inequalities, rather than to promote health interventions that merely target individuals or populations that are affected by such inequalities.

- Thank you. This is a great point. The text has been updated to clarify between addressing structural inequalities and mitigating impacts among those most affected. The text now reads: “With locations in East Harlem, Brownsville, and Tremont, and with many established community partners, the Neighborhood Health Action Centers, while not positioned to address the structural causes of inequalities described, can be useful tools in mitigating gentrification-associated health outcomes in the most impacted communities (65). Further NYC DOHMH gentrification research should include investigating health impacts of policies that directly address structural inequalities.”

**Acknowledgements:**

Check the spelling of Kathleen Reilly’s name

- It was spelled incorrectly and has been fixed, thank you for catching this!

**Reviewer #2:** I am not familiar with the exact methods the author's used (joinpoint regression) so I cannot answer whether the methods were appropriate. In general the study addresses an important topic. I think contextualizing the findings and the motivation for the study is an important way the paper can be improved.

- Thank you for this feedback. We have helped to contextualize the findings by expanding on the description of the type of gentrification that was occurring during the study period. We wrote “Later-stage gentrification (the third through fifth waves) have been driven by money-making ventures and characterized by urban commodification (13). A recent analysis of NYC real estate indicated that in 2003 most building purchases were made by individuals, but by 2014 corporations represented the predominant property buyer in nearly all neighborhoods (14). Landlords that own multiple buildings, such as corporations, have a higher tenant eviction rate per unit compared with landlords who own few units (15). This study focuses on neighborhoods that experienced later-stage gentrification occurring during 2000–2017.
- In addition, we also clarified the motivation behind these analyses: to better understand how gentrification might contribute to health inequities and ultimately to improve upon the services provided by the NYC Health Department. We wrote “To improve resiliency and reduce mental health disparities in the face of gentrification, policy makers can consider interventions that strengthen safeguards for tenants such as offering legal support for those facing eviction or harassment, and financial assistance for rent; increase access to subsidized housing allowing original residents to remain in their neighborhoods without fear of displacement; and partnerships with community organizations to promote political empowerment for all residents (37). Future neighborhood developments can engage community residents and offer decision making power to create a vision for neighborhood change that is shared among all residents. The NYC Department of Health and Mental Hygiene, through the Neighborhood Health Action Centers, has mechanisms in place and physical space to implement such community interventions to neighborhoods where gentrification is currently taking place. With locations in East Harlem, Brownsville, and Tremont, and with many established community partners, the Neighborhood Health Action Centers, while not positioned to address the structural causes of inequalities described, can be useful tools in mitigating gentrification-associated health outcomes in the most impacted communities (65). Further NYC DOHMH gentrification research should include investigating health impacts of policies that directly address structural inequalities.”

When submitting your revision, we need you to address these additional requirements.

1. Please ensure that your manuscript meets PLOS ONE's style requirements, including those for file naming. The PLOS ONE style templates can be found at 
https://journals.plos.org/plosone/s/file?id=wjVg/PLOSOne_formatting_sample_main_body.pdf and 
https://journals.plos.org/plosone/s/file?id=ba62/PLOSOne_formatting_sample_title_authors_affiliations.pdf

2. In your Data Availability statement, you have not specified where the minimal data set underlying the results described in your manuscript can be found. PLOS defines a study's minimal data set as the underlying data used to reach the conclusions drawn in the manuscript and any additional data required to replicate the reported study findings in their entirety. All PLOS journals require that the minimal data set be made fully available. For more information about our data policy, please see http://journals.plos.org/plosone/s/data-availability.

Upon re-submitting your revised manuscript, please upload your study’s minimal underlying data set as either Supporting Information files or to a stable, public repository and include the relevant URLs, DOIs, or accession numbers within your revised cover letter. For a list of acceptable repositories, please see http://journals.plos.org/plosone/s/data-availability#loc-recommended-repositories. Any potentially identifying patient information must be fully anonymized.

Important: If there are ethical or legal restrictions to sharing your data publicly, please explain these restrictions in detail. Please see our guidelines for more information on what we consider unacceptable restrictions to publicly sharing data: http://journals.plos.org/plosone/s/data-availability#loc-unacceptable-data-access-restrictions. Note that it is not acceptable for the authors to be the sole named individuals responsible for ensuring data access.

We will update your Data Availability statement to reflect the information you provide in your cover letter.

- The majority of the data used in this manuscript can be found in the Open Science Framework Data Repository at <https://osf.io/szv36>. The repository contains a document titled: Gentrifcation_Manuscript_Datasets.doc that explains the contents of the files and original data sources for the Census and American Community Survey data used to classify neighborhoods into gentrification categories and Census Bureau population estimates.
- The Community Health Survey data used to estimate the prevalence of serious psychological distress are available, albeit limited, at <https://www1.nyc.gov/site/doh/data/data-sets/community-health-survey-public-use-data.page>. The Community Health Survey Public Use Data is available at the city-wide level. In order to access the UHF level data as used in the manuscript, you would need to contact [epidatarequest@health.nyc.gov](mailto:epidatarequest@health.nyc.gov) to arrange a Data Use Agreement.

3. We note that Figure 1 in your submission contain [map/satellite] images which may be copyrighted. All PLOS content is published under the Creative Commons Attribution License (CC BY 4.0), which means that the manuscript, images, and Supporting Information files will be freely available online, and any third party is permitted to access, download, copy, distribute, and use these materials in any way, even commercially, with proper attribution. For these reasons, we cannot publish previously copyrighted maps or satellite images created using proprietary data, such as Google software (Google Maps, Street View, and Earth). For more information, see our copyright guidelines: http://journals.plos.org/plosone/s/licenses-and-copyright.
 
We require you to either (1) present written permission from the copyright holder to publish these figures specifically under the CC BY 4.0 license, or (2) remove the figures from your submission:
a. You may seek permission from the original copyright holder of Figure 1 to publish the content specifically under the CC BY 4.0 license.  
We recommend that you contact the original copyright holder with the Content Permission Form (http://journals.plos.org/plosone/s/file?id=7c09/content-permission-form.pdf) and the following text:
“I request permission for the open-access journal PLOS ONE to publish XXX under the Creative Commons Attribution License (CCAL) CC BY 4.0 (http://creativecommons.org/licenses/by/4.0/). Please be aware that this license allows unrestricted use and distribution, even commercially, by third parties. Please reply and provide explicit written permission to publish XXX under a CC BY license and complete the attached form.”
 
Please upload the completed Content Permission Form or other proof of granted permissions as an "Other" file with your submission.
 
In the figure caption of the copyrighted figure, please include the following text: “Reprinted from [ref] under a CC BY license, with permission from [name of publisher], original copyright [original copyright year].”
 
b. If you are unable to obtain permission from the original copyright holder to publish these figures under the CC BY 4.0 license or if the copyright holder’s requirements are incompatible with the CC BY 4.0 license, please either i) remove the figure or ii) supply a replacement figure that complies with the CC BY 4.0 license. Please check copyright information on all replacement figures and update the figure caption with source information. If applicable, please specify in the figure caption text when a figure is similar but not identical to the original image and is therefore for illustrative purposes only.
The following resources for replacing copyrighted map figures may be helpful:
 
USGS National Map Viewer (public domain): http://viewer.nationalmap.gov/viewer/
The Gateway to Astronaut Photography of Earth (public domain): http://eol.jsc.nasa.gov/sseop/clickmap/
Maps at the CIA (public domain): https://www.cia.gov/library/publications/the-world-factbook/index.html and https://www.cia.gov/library/publications/cia-maps-publications/index.html
NASA Earth Observatory (public domain): http://earthobservatory.nasa.gov/
Landsat: http://landsat.visibleearth.nasa.gov/
USGS EROS (Earth Resources Observatory and Science (EROS) Center) (public domain): http://eros.usgs.gov/#
Natural Earth (public domain): <http://www.naturalearthdata.com/>

- We own Figure 1 and it is not copyrighted by another entity. This figure was created using a UHF shapefile that is produced by the NYC DOHMH and available for public use. It can be found here: <https://www1.nyc.gov/site/doh/data/data-sets/maps-gis-data-files-for-download.page> and in the Open Science Framework Data Repository at <https://osf.io/szv36>. The other data used to create this figure were the Census and ACS data described in the manuscript and in the repository. The figure was created using just these data sets and a combination of two opensource software: R and QGIS.
