# Supplementary material for: Can changing neighborhoods influence mental health? An ecological analysis of gentrification and neighborhood-level serious psychological distress—New York City, 2002–2015
Source: PLoS One. 2023 Apr 5;18(4):e0283191. doi: 10.1371/journal.pone.0283191 (PMC10075454; doi:10.1371/journal.pone.0283191)
Supplement: Attachment — Submitted filename: Response to reviewers.docx [file pone.0283191.s004.docx]

Please see below for a point-by-point response to the reviewers’ comments. Line numbers reference the clean (non-track changes) version of the resubmitted manuscript.

Reviewer's Responses to Questions

**Comments to the Author**

1. If the authors have adequately addressed your comments raised in a previous round of review and you feel that this manuscript is now acceptable for publication, you may indicate that here to bypass the “Comments to the Author” section, enter your conflict of interest statement in the “Confidential to Editor” section, and submit your "Accept" recommendation.

Reviewer #1: All comments have been addressed

Reviewer #2: (No Response)

Reviewer #3: (No Response)

2. Is the manuscript technically sound, and do the data support the conclusions?

The manuscript must describe a technically sound piece of scientific research with data that supports the conclusions. Experiments must have been conducted rigorously, with appropriate controls, replication, and sample sizes. The conclusions must be drawn appropriately based on the data presented.

Reviewer #1: Yes

Reviewer #2: Partly

Reviewer #3: Partly

3. Has the statistical analysis been performed appropriately and rigorously?

Reviewer #1: Yes

Reviewer #2: Yes

Reviewer #3: Yes

4. Have the authors made all data underlying the findings in their manuscript fully available?

The [PLOS Data policy](https://urldefense.com/v3/__http:/www.plosone.org/static/policies.action*sharing__;Iw!!MXfaZl3l!bDR07v73btojPOuZ4a1p5f13MOyjOI2c-le02Df92ihlTLl7wEVqWdktNMoAvMeJpwMv5dcRgP2OoWgOL975tOE5bw$) requires authors to make all data underlying the findings described in their manuscript fully available without restriction, with rare exception (please refer to the Data Availability Statement in the manuscript PDF file). The data should be provided as part of the manuscript or its supporting information, or deposited to a public repository. For example, in addition to summary statistics, the data points behind means, medians and variance measures should be available. If there are restrictions on publicly sharing data—e.g. participant privacy or use of data from a third party—those must be specified.

Reviewer #1: Yes

Reviewer #2: Yes

Reviewer #3: No

5. Is the manuscript presented in an intelligible fashion and written in standard English?

PLOS ONE does not copyedit accepted manuscripts, so the language in submitted articles must be clear, correct, and unambiguous. Any typographical or grammatical errors should be corrected at revision, so please note any specific errors here.

Reviewer #1: Yes

Reviewer #2: Yes

Reviewer #3: Yes

6. Review Comments to the Author

Please use the space provided to explain your answers to the questions above. You may also include additional comments for the author, including concerns about dual publication, research ethics, or publication ethics. (Please upload your review as an attachment if it exceeds 20,000 characters)

Reviewer #1: The authors have addressed all comments satisfactory. I only have one remaining concern.

Regarding the use of quotes from participants in an external publication, I still think these should be removed. While I understand the authors' intent of representing community perspectives which could not be captured by the quantitative data analyzed for the study, what should be presented here is the relevant findings from those external studies as interpreted by the authors of the external studies. To take a quotation from the results section of a qualitative study and publish it separately takes the citation away from the context in which it was analyzed. To use a piece of data like this also may violate research ethics in that the participant whose quote it is would have given permission to those authors to use their quote anonymously in their article. But they would not have given permission for the quote to then be reproduced elsewhere. Therefore, I would like to insist that these quotes be removed.

Thank you for this feedback, the reviewer makes a good point. The two direct quotes from qualitative research studies were removed from the manuscript.

Reviewer #2: The authors did a great job engaging with the previous reviewer feedback. I was quite impressed. I have a few comments and suggestions that the authors should consider.

Thank you very much for your review and support, we truly think this helped to improve the manuscript.

Many of the references about gentrification and community are older. The authors should review recent research by Joseph Gibbons and colleagues to these references.

Thank you for this recommendation, we have included updated references by author Joseph Gibbons and colleagues, including the following two references:

- Gibbons J. Are gentrifying neighborhoods more stressful? A multilevel analysis of self-rated stress. SSM-population health. 2019 Apr 1;7:100358.
- Gibbons J, Barton MS, Reling TT. Do gentrifying neighbourhoods have less community? Evidence from Philadelphia. Urban Studies. 2020 May;57(6):1143-63.

The statement that gentrification measure is debated needs a citation. The authors should consider Barton (2016 Urban Studies) and Brown-Saracino (2017 Annual Review).

Thank you for these recommended citations, these two references were added accordingly.

Related, given the variation in measurement, why was this particular strategy used? Were any other measures assessed to check for sensitivity? Barton and Cohen (Social Science Research) show that the measurement strategy has important implications. I don't think a second strategy needs to be reported, but the authors should engage with this issue in a slightly more thorough fashion.

On line 98, we have included additional rationale for the selection of the gentrification measure used in this study “We selected the Furman Center definition to build on this Center’s substantial foundation of scholarship on gentrification in NYC, and because the definition relied on multiple economic factors. Gentrification definitions that incorporate social criteria, such as educational attainment and single-parent households, might unfairly put the onus of neighborhood change on the neighborhood’s community members.”

The materials and methods section was underdeveloped. This could be expanded just a little to highlight/remind the reader of how this manuscript contributes to research on gentrification with health.

Thank you for this suggestion. Initially in the abstract on line 57 we had written “This analysis highlights potential unequal mental health impacts that can be associated with gentrification-related neighborhood changes.“ However, we completely agreed that we needed to more clearly highlight how this manuscript contributes to research on gentrification and health.

To rectify this, in the introduction we added a section to more carefully describe our aims in order to help clarify and contextualize our methods section. On line 123 we wrote “In this study we aimed to characterize trends in gentrification and neighborhood-level serious psychological distress over time, and how the relationship varied by racial/ethnic populations. Given the relatedness between racism and gentrification reported in previous literature (28), we hypothesized that the population composition of original residents versus new residents, and thus who are exposed to gentrification, varied by race/ethnicity. We explored the validity of this assumption in an ancillary analysis using housing mobility data”

Lastly we included additional language in the Discussion section which highlights how our findings contribute to the literature. We have added at line 360 the following text  “These findings enhance our understanding of gentrification and health by showing how neighborhood population mental health in NYC has changed over time as neighborhoods themselves have changed, and by reinforcing the notion that gentrification has differential racial/ethnic impacts and can exacerbate health disparities.”

I understand the challenge of studying gentrification in New York City. In particular, having to use larger units of analysis such as UHFs or sub-boroughs. UHFs are large areas and gentrification typically occurs in a small part of a neighborhood. Barton (Crime and Delinquency) looked at the percent of tracts within subboroughs that gentrified. It's not a great measure, but it is more precise than simply looking at gentrification in the larger units. In short, there needs to be more discussion of the units of analysis to better justify these units and how gentrification is measured.

Thank you for pointing this out, it is a great point. While the gentrification method described in the Barton paper is smart and may provide more granularity within the bigger geography, the NYC DOHMH often uses UHF for community and neighborhood health metrics because this geography has meaningful neighborhood boundaries. The text has been updated in the discussion to reflect this.  Lines 492-505 now state “Additionally, the gentrification exposure might not be acting uniformly within neighborhoods, and some neighborhoods might be influenced by gentrification at different times, speeds, and spatial scales. The causally relevant geographic unit important for mental health outcomes is unknown, and mental health may also be influenced by the neighborhoods where people work, attend school, or otherwise spend time aside from their residential neighborhood. Thus, the study is susceptible to the uncertain geographic context problem and spatial polygamy, respectively (65). We examined gentrification at the UHF level, as this is the smallest geography for which our survey data can provide representative prevalence estimates, however, since gentrification can occur on a hyper-local spatial scale, gentrification might be heterogeneous within a given UHF (Fig 1).  Nonetheless, UHFs, and the Community Districts they were designed to approximate, represent meaningful neighborhood boundaries. Residents in these neighborhoods share community board members who advise important decisions on resource allocation, land use, zoning, and other city planning and development projects, all of which are relevant to gentrification (29, 66).”

The first paragraph of the discussion should include a reminder of how this study contributed to previous research. This is a minor issue, but it helps with the flow of the manuscript.

Thank you for this suggestion, we have added a sentence at the end of the first paragraph of the discussion that clarifies how our work contributes to prior research. On line 360 we say “These findings enhance our understanding of gentrification and health by showing how neighborhood population mental health in NYC has changed over time as neighborhoods themselves have changed, and by reinforcing the notion that gentrification has differential racial/ethnic impacts and can exacerbate health disparities.”

Overall, I think this is an interesting study and has the potential to make contributions to research on gentrification and health. The authors did a great job engaging with the previous reviewer feedback and I hope they find these comments helpful.

Thank you so much!

Reviewer #3: This is a revised manuscript (MS) aiming to examine serious psychological distress as an indicator of gentrification’s impact on mental health in New York City (NYC) and whether the effect on mental health varies with race/ethnicity. To address this aim, the authors used data from two sources: The NYC Community Health Survey (CHS; years 2002, 2003, 2005, 2006, 2008, 2009, 2010, 2012, 2013, and 2015) and the American Community Survey (5-year estimates: 2013-2017).

Specific aims:

1) The abstract should provide the complete aim of the paper, i.e., variation by race/ethnicity.

Thank you for this recommendation. In the abstract on lines 39-42 we revised our stated aim by saying “To ultimately target health promotion interventions, we examined serious psychological distress time trends in gentrifying NYC neighborhoods to describe the association of gentrification and mental health overall and stratified by race and ethnicity.“

2) The study is ecological as per the title. However, the language in the abstract goes back and forth from individuals to populations (lines 50-54).

Thank you for this recommendation. Here in the abstract, and throughout the manuscript language was revised to ensure that analyses reflected neighborhood-level population estimates and not estimates of individuals.

3) The introduction does not provide a rationale for the use of psychological distress or why a difference by race/ethnicity should be expected.

Thank you for these comments.

**Psychological distress:** Lines 104-105 state “We chose to examine serious psychological distress as an indicator of gentrification’s impact on mental health based on literature evidence and mechanistic plausibility (12, 21-24).” We have added at lines 105-107 “We chose to use serious psychological distress as it captures a variety of mental health disorders in a single metric at a population level (25).

**Difference by race/ethnicity:** Throughout the introduction we describe how gentrification may intersect with race and ethnicity and how the practices creating gentrified neighborhoods are deeply embedded in racism. We have added (lines 115-117) to emphasize how this informed our methods where we state “We examined serious psychological distress stratified by race/ethnicity, as we expected mental health outcomes related to gentrification to vary by race”. Which is followed by examples in lines 117-121 about exclusion and displacement related to race. We have additionally added at line 124 “Given the relatedness between racism and gentrification reported in previous literature (28), we hypothesized that the population composition of original residents versus new residents, and thus who are exposed to gentrification, varied by race/ethnicity.”

4) The aims could be more clearly stated as they are lost in the last paragraph of the introduction (lines 93-108).

Thank you for this recommendation, for increased clarity we added the following text at lines 123-128 “In this study we aimed to characterize trends in gentrification and neighborhood-level serious psychological distress over time, and how the relationship varied by racial/ethnic populations. Given the relatedness between racism and gentrification reported in previous literature (28), we hypothesized that the population composition of original residents versus new residents, and thus who are exposed to gentrification, varied by race/ethnicity. We explored the validity of this assumption in an ancillary analysis using housing mobility data.”

5) The methods section has an unusual presentation and is hard to follow. For instance, the description of the datasets are combined with the descriptions of the exposure and outcome.

While we understand where the reviewer is coming from, we chose to describe the data sets with the exposure and outcome variable definitions, as separate data sets were used for each metric. Typically, if the exposure and outcome were coming from the same data set, we would describe the data set first and then dive into exposure and outcome variables separately.  In this specific situation, this presentation would be confusing and require us to repeat ourselves in sections about data sets and variables. Given the limited character space, we respectfully choose to keep the methods how they currently are presented.

6) There is no justification for the years of the data included for the outcome.

We added a sentence to line 176 in the methods section that clarified that “The timeframe examined in this study was, in part, shaped by the available outcome data on psychological stress.”

In addition, we added a sentence in the abstract on line 45 that clarifies that “To temporally align neighborhood categorization closely with neighborhood-level measurement of serious psychological distress, data during 2000–2017 were used to classify neighborhood type.”

7) The statistical analysis section could be more succinct and better organized to reflect the aims. In fact, as is, the statistical analysis description seems disconnected from the aims: there was never mentioning of trend over time or housing mobility in the aims.

Thank you for pointing this out. We reordered the sentences in the section on statistical analysis so that it first describes the analysis on time trends, linking to our primary aim. On line 248 the text reads “For time trend analyses, statistical significance of trends was determined if regression model estimate P-values were <0.05.”

After this, in the second sentence of this statistical analysis paragraph, we describe the statistical analysis related to our secondary aim which examined housing mobility. On line 249 we wrote “Pairwise T-tests were used to compare different strata using a reference category for the 2015 Community Health Survey data on serious psychological distress, and 2017 Community Health Survey data on housing mobility.”

Also in the methods, words/phrases were reworked or deleted to be more succinct in. For example, in the section describing time trend analyses we removed “also known as change point or segmented regression”.

We hope that these changes, in conjunction with the more clearly articulated aims in the introduction in lines 123-128 will help readers more easily understand our methodology. Now our aims are written as follows “In this study we aimed to characterize trends in gentrification and neighborhood-level serious psychological distress over time, and how the relationship varied by racial/ethnic populations. Given the relatedness between racism and gentrification, we reasonably assumed that the population composition of original residents versus new residents, and thus who are exposed to gentrification, varied by race/ethnicity. We explored the validity of this assumption in an ancillary analysis using housing mobility data.”

8) Why wasn’t there a Table 1 describing details for the datasets used, i.e., number of neighborhood gentrified, mean (median) number of people per neighborhoods, prevalence of psychological distress by neighborhoods, demographics of the neighborhoods based on gentrification, etc.?

Thank you for this recommendation. The geography we used in our analysis was the United Hospital Fund (UHF), which is described in our manuscript on line 152 “as 42 adjoining zip code areas intended to approximate NYC community planning districts, with roughly 200,000 individuals residing within each UHF.“ We added an additional sentence on line 153 directing readers to the community profile websites in case they sought out additional neighborhood information “Detailed neighborhood profiles are described online by the NYC Health Department.” These neighborhood profiles have been created by the NYC Department of Health and Mental Hygiene and they describe in detail the characteristics and health metrics for each NYC neighborhood. These can be found here: <https://www.nyc.gov/site/doh/data/data-publications/profiles.page>

We originally summarized the neighborhood characterization in writing in the results section and graphically in Figure 1. The geographic illustration offered meaningful context both for people familiar with and those unfamiliar with NYC neighborhoods. Stemming from this recommendation, however, we have created an S1 Table as supplementary material for those readers who want to see additional detail on how each neighborhood met the different categorization criteria.

9) Where is the data presented in lines 248-256? If not in tables, please say so.

We added a new table describing the criteria for neighborhood classification and it was added to the supplementary materials. On line 264 we revised the text to read “A summary of the gentrification criteria by neighborhood can be found in the supplementary material in S1 Table.“

10) Figure 3 needs legends for the axes.

Given that there can be subtle differences in the seven shades of gray used in this figure, we felt that directly labeling the bar graphs would be easier for readers to interpret than creating a legend.

11) The Discussion section is too long and a bit out of scope of the aims.

Thank you for this feedback. We have addressed this recommendation in multiple ways.

- We removed quotes from participants of qualitative research, as these were not referencing any of the aims we stated.
- We updated the aims in the introduction section to more explicitly include the housing mobility analysis. This brings greater relevance of the issue of housing mobility described in the 4th paragraph of the discussion.
- Other sentences were removed that we thought were less relevant to specific aims. These include the sentence reading “Older adults living in neighborhoods undergoing gentrification have more symptoms of depression and anxiety compared with counterparts in high-income neighborhoods”

12) The limitations should include the effect of MAUP, uncertainty geographic context problem and spatial polygamy.

Thank you for pointing this out. Lines 489-498 now include a discussion of these concepts.

“An ecological study design inherently limits inferences to an aggregated neighborhood-level, and we cannot infer causality of gentrification to individual-level health outcomes in this study. Related, the modifiable areal unit problem, also results from the aggregation of survey data to UHF geography, as the aggregate values are influenced by what geography is chosen (64). Additionally, the gentrification exposure might not be acting uniformly within neighborhoods, and some neighborhoods might be influenced by gentrification at different times, speeds, and spatial scales. The causally relevant geographic unit important for mental health outcomes is unknown, and mental health may also be influenced by the neighborhoods where people work, attend school, or otherwise spend time aside from their residential neighborhood. Thus, the study is susceptible to the uncertain geographic context problem and spatial polygamy, respectively (65).”

7. PLOS authors have the option to publish the peer review history of their article ([what does this mean?](https://urldefense.com/v3/__https:/journals.plos.org/plosone/s/editorial-and-peer-review-process*loc-peer-review-history__;Iw!!MXfaZl3l!bDR07v73btojPOuZ4a1p5f13MOyjOI2c-le02Df92ihlTLl7wEVqWdktNMoAvMeJpwMv5dcRgP2OoWgOL97WV1dmpQ$)). If published, this will include your full peer review and any attached files.

If you choose “no”, your identity will remain anonymous but your review may still be made public.

**Do you want your identity to be public for this peer review?** For information about this choice, including consent withdrawal, please see our [Privacy Policy](https://urldefense.com/v3/__https:/www.plos.org/privacy-policy__;!!MXfaZl3l!bDR07v73btojPOuZ4a1p5f13MOyjOI2c-le02Df92ihlTLl7wEVqWdktNMoAvMeJpwMv5dcRgP2OoWgOL94ZrV5xxA$).

Reviewer #1: No

Reviewer #2: No

Reviewer #3: No
