# Supplementary material for: Can changing neighborhoods influence mental health? An ecological analysis of gentrification and neighborhood-level serious psychological distress—New York City, 2002–2015
Source: PLoS One. 2023 Apr 5;18(4):e0283191. doi: 10.1371/journal.pone.0283191 (PMC10075454; doi:10.1371/journal.pone.0283191)
Supplement: Attachment — Submitted filename: Response to Reviewers.docx [file pone.0283191.s005.docx]

Please see below a point-by-point response to editor/reviewer comments for our article submission, PONE-D-21-39108R2, Can changing neighborhoods influence mental health? An ecological analysis of gentrification and neighborhood-level serious psychological distress — New York City, 2002–2015.

Dear Dr. Cavalier,

Thank you for submitting your manuscript to PLOS ONE. After careful consideration, we feel that it has merit but does not fully meet PLOS ONE’s publication criteria as it currently stands. Therefore, we invite you to submit a revised version of the manuscript that addresses the points raised during the review process.

There are several remaining concerns raised by reviewers that should be addressed in full. Concerning the removal of quotations, this depends entirely upon both (a) whether study participants agreed to be directly quoted in materials resulting from the study and (b) whether the appropriate ethics approvals including a detailed description of the qualitative data collection and data handling were secured, if applicable at your institute. If both of these criteria are fulfilled, please do retain the quotes, as they provide interesting contextual data; however, both points (a) and (b) should be explicitly addressed in the revised manuscript text.

We believe that the reviewer who suggested the removal of the quotes made a compelling argument that it is not standard practice to include participant quotes from other studies, and that this practice removes the quote from the rich context the qualitative study provides. Thus, we do not feel inclined to attempt to contact the original authors and request the additional ethics approvals needed to keep the quotes in our paper.

Journal Requirements:

Please review your reference list to ensure that it is complete and correct. If you have cited papers that have been retracted, please include the rationale for doing so in the manuscript text, or remove these references and replace them with relevant current references. Any changes to the reference list should be mentioned in the rebuttal letter that accompanies your revised manuscript. If you need to cite a retracted article, indicate the article’s retracted status in the References list and also include a citation and full reference for the retraction notice.

Thank you for your close attention to our reference list. We are grateful for this feedback, because these corrections help to attribute information more accurately to current gentrification and health literature. We have made the following changes to ensure appropriateness and accuracy of citations.

- In the discussion on line 374, reference 43 was corrected to reference 42 (Brummet et al) – this paper indicates that some original residents might benefit from neighborhood gentrification. No downstream numbering was impacted due to this change, because reference 43 (Izenberg et al) was cited again in the same sentence – a paper that describes that gentrification can harm original residents.
- The Anguelovski reference (originally reference 57) was removed because people in Barcelona are not considered Latinos (in general Spanish speaking from Spain are considered Hispanic, but we had another reference already for this sentence – reference 56- and we thought it would be more confusing to keep reference 57 and/or highlight this difference)
- The Glaeser reference (originally reference 61) was removed. This online report posted by the National Bureau of Economic Research was very recently revised in February 2023. The report no longer includes the example about the Boyle Heights LA neighborhood, that we had originally included in our discussion. To make our point that gentrification solely based on economics does not account for certain psychosocial changes we site other references, including a review article by Bhavsar et al (now reference 59), as well as Lance Freeman’s book ‘There goes the hood: Views on gentrification from the ground up’ (now reference 60).

[Note: HTML markup is below. Please do not edit.]

Reviewers' comments:


[NOTE: If reviewer comments were submitted as an attachment file, they will be attached to this email and accessible via the submission site. Please log into your account, locate the manuscript record, and check for the action link "View Attachments". If this link does not appear, there are no attachment files.]

While revising your submission, please upload your figure files to the Preflight Analysis and Conversion Engine (PACE) digital diagnostic tool, [https://pacev2.apexcovantage.com/](https://urldefense.com/v3/__https:/pacev2.apexcovantage.com/__;!!MXfaZl3l!dANR0peBEcLwOTDMK1a-bFfk-TWyXnzvSvdPaOk2qsmVmk-TNmRBJ6rzlL2d1jVu0VpT1beTJwUgn-HVl7g-5ztaCw$). PACE helps ensure that figures meet PLOS requirements. To use PACE, you must first register as a user. Registration is free. Then, login and navigate to the UPLOAD tab, where you will find detailed instructions on how to use the tool. If you encounter any issues or have any questions when using PACE, please email PLOS at [figures@plos.org](mailto:figures@plos.org). Please note that Supporting Information files do not need this step.

Done. PACE made a few adjustments to the figures including flattening, compressing, adjusting dimensions, and removing the Alpha channel. The adjusted figures were downloaded from PACE and will be the files submitted with this revision.
